# Transcriptomic analysis of *Hericium erinaceus* mycelia at distinct growth stages: insights into molecular mechanisms underlying mycelial proliferation

**DOI:** 10.3389/ffunb.2026.1778644

**Published:** 2026-08-18

**Authors:** Hailong Zong, Yuhan Duan, Xiaolei Li, Hongchun Bao

**Affiliations:** 1 Agricultural College, Inner Mongolia Agricultural University, Hohhot, China; 2 Agricultural and Animal Husbandry Technology Extension Center of Inner Mongolia Autonomous Region, Hohhot, China

**Keywords:** amino acid metabolism, differential expression gene, *Hericium erinaceus*, mycelial growth, transcriptomics

## Abstract

*Hericium erinaceus*, a highly valued edible fungus, is among the most widely consumed mushrooms globally, attributed to its superior nutritional composition and delicate organoleptic properties. Extensive prior studies have consistently shown that the yield of mature *H. erinaceus* fruiting bodies is tightly linked to mycelial growth status. Mycelia serve as the essential vegetative structure that supports nutrient acquisition and subsequent reproductive development. To decipher the molecular mechanisms regulating *H. erinaceus* mycelial growth, transcriptome profiling was conducted on mycelial samples from strains with divergent growth rates, harvested at distinct growth stages. Transcriptomic analysis identified 3,257 DEGs across the two genotypic groups. Notably, upregulated DEGs outnumbered downregulated ones significantly, indicating a predominantly activated transcriptional program during mycelial growth. GO functional annotation revealed these DEGs were primarily enriched in terms related to oxidoreductase activity, which is an enzymatic function critical for mediating redox reactions in energy metabolism and secondary metabolite biosynthesis. KEGG pathway enrichment analysis further showed that most DEGs were concentrated in amino acid metabolism pathways, including arginine and proline metabolism, phenylalanine metabolism, and tryptophan metabolism. These pathways are pivotal for supplying essential nitrogen sources, regulating cellular osmotic balance, and synthesizing bioactive molecules that support mycelial proliferation. Collectively, this study generates comprehensive transcriptomic data for *H. erinaceus* mycelia, which not only offer valuable insights into the molecular mechanisms driving mycelial growth but also facilitate the identification of key candidate genes regulating mycelial development. These findings establish a robust theoretical basis for optimizing stable large-scale cultivation of *H. erinaceus* and advancing molecular-assisted breeding to improve its agronomic traits.

## 1 Introduction

Edible fungi, a group of edible macrofungi, have evolved into an indispensable component of daily diets in China and other Eastern nations (Grimm and Wösten, 2018). Notably, they play a pivotal role in ecological, agricultural, industrial, and medicinal sectors by converting lignocellulosic agricultural residues into food, feed, and organic fertilizers (Chang and Buswell, 2022). As a sustainable and nutritionally valuable food source, edible fungi also possess prominent medicinal properties, as numerous nutrients and bioactive compounds, such as high-quality proteins and polysaccharides, can be isolated from their mycelia and fruiting bodies, thereby facilitating their extensive application in functional health foods (Dong et al., 2022). Regarded as one of the fastest-growing organisms globally, edible fungi undergo three sequential developmental stages, including the basidiospore stage, the mycelium stage, and the fruiting body stage (Chukwu et al., 2022). As the nutritional organ of edible fungi, mycelium absorbs nutrients, water, inorganic salts, and organic compounds to sustain its growth and development, while also contributing significantly to biogeochemical cycles (Okuda, 2022). Specifically, mycelium can directly take up simple carbohydrates and organic acids; in contrast, for complex macromolecular substances such as cellulose, hemicellulose, and lignin, it must secrete extracellular enzymes to decompose these polymers into absorbable monosaccharides prior to uptake (Zhao et al., 2016). Following nutrient absorption, spores of edible fungi elongate along their longitudinal axis to form villus-like primary hyphae, which further develop into filamentous secondary hyphae under favorable environmental conditions that are capable of differentiating into fruiting bodies (Feng et al., 2024). Subsequent development gives rise to cord-like tertiary hyphae with the potential to differentiate into various tissues. Through continuous division, elongation, and branching, these hyphae intertwine to form a compact mycelial network, which then twists and differentiates into fruiting body primordia, ultimately maturing into edible fruiting bodies (Liu et al., 2024a).

*Hericium erinaceus* (Bull.) Pers., also known as lion’s mane mushroom, monkey’s head mushroom, or Yamabushitake, is a valuable and widely cultivated edible fungus that contains abundant essential nutrients including protein, dietary fiber and carbohydrates (Gong et al., 2020). Furthermore, the lion’s mane mushroom contains a high concentration of active components such as polysaccharides, polyphenols, and flavonoids, making it both nutritionally and medicinally valuable (Yanshree et al., 2022). It is found all throughout China and has a wealth of natural resources. The Greater and Lesser Khingan Mountains in the northeast, the Tianshan Mountains in the northwest, the Himalayas in the west, the Hengduan Mountains in the southwest, the Altai Mountains, and other forested regions are among its primary distribution areas. The greatest of the valuables is the monkey head mushroom, which grows in the Great Xing’an Mountain virgin forest (Jahedi et al., 2024).

During the growth and development of edible fungi, faster mycelial growth rates more effectively reflect the fungus’s environmental adaptability, as they indicate higher efficiency in the absorption and conversion of nutrients from the culture medium, stronger physiological metabolic activity, and more robust overall growth vitality (Chen et al., 2025). Furthermore, variations in mycelial growth rate directly mirror the fungus’s capacity to acclimate to its surroundings. Biomass refers to the total mass of all viable cultures within the medium, and higher biomass denotes more vigorous mycelial growth. Proteins, soluble sugars, and proline are core physiological metabolites. Fluctuations in their concentrations directly reflect the mycelium’s nutritional status and metabolic activity, and thus serve as key biochemical indicators for evaluating mycelial growth vigor (Diamantopoulou and Papanikolaou, 2023; Alvarez et al., 2022). Mycelia secrete extracellular enzymes to degrade and assimilate nutrients from the culture medium, and these enzymes facilitate the conversion of the nutrients into the fungus’s own proteins and polysaccharides. Notably, the level of extracellular enzyme activity at a specific developmental stage can also reflect the intensity of mycelial vitality. Consequently, the cellulase activity level in edible mushroom mycelia indicates the extent to which mycelia absorb and utilize nutrients from the culture medium, and this provides important insights for investigating mycelial growth during a given period (Zhou et al., 2018).

With the development of sequencing technology, the wide application of genomics in the field of biology has been greatly promoted (Lodi et al., 2025). In addition, Transcriptome sequencing method can fully identify all actively transcribed gene products in particular cells, tissues, or organisms at various stages of development, including mRNA and non-coding RNA (Liu et al., 2024b). In macrofungal research, transcriptome-based studies have been carried out on various species, including *Flammulina velutipes* (Yang et al., 2018), *Agrocybe cylindracea* (Wang et al., 2021), *Lentinula edodes* (Sakamoto et al., 2017), *Ganoderma lucidum* (Meng et al., 2022), and *Cordyceps militaris* (Luo et al., 2021). Using RNA sequencing (RNA-seq), transcriptomic profiling has been performed on the hyphae and fruiting bodies of *F. velutipes*, *A. cylindracea*, *G. lucidum*, and *A. polytricha* to identify differentially expressed sequence tags (DESTs), evaluate tissue-specific gene expression patterns, and assess transcriptional conservation during growth and development (Yan et al., 2020). These efforts have laid the groundwork for fundamental research on the molecular mechanisms underlying hyphal pigmentation in *L. edodes* and the gene expression dynamics across different developmental stages of *C. militaris*. Liu et al. (2019) conducted a comprehensive transcriptomic analysis of *Morchella* importuna at the vegetative mycelium (VM), initial sclerotium (IS), and mature sclerotium (MS) stages using deep RNA sequencing and *de novo* transcriptome assembly. Their results revealed that carbohydrate catabolism was predominant in the vegetative mycelium stage, whereas anabolism of high-energy metabolites constituted the primary metabolic signature underlying both mycelial growth and sclerotial morphogenesis in *M*. *importuna*. In parallel, Hao et al. (2019) performed transcriptomic profiling of *M*. *importuna* at the mycelial and young fruiting body stages and demonstrated that genes encoding carbohydrate-active enzymes (CAZymes), mitochondrial proteins, oxidoreductases, and heat shock proteins were significantly upregulated in young fruiting bodies relative to mycelia.

Currently, research on the monkey head mushroom remains focused primarily on optimizing cultivation techniques and evaluating the bioactivity of crude extracts, with few reports on the mechanisms regulating its growth and development. Therefore, to characterize the molecular basis governing mycelial growth in H. erinaceus, comparative transcriptome analysis was performed across two critical stages of mycelial growth to screen for key candidate genes involved in the regulation of mycelial development. This work provides novel insights into the differential growth characteristics of H. erinaceus strains and reveals putative regulatory patterns of growth-related genes. The results serve as an important reference for subsequent functional validation, as well as for cultivation optimization and molecular breeding of this edible fungus.

## 2 Materials and methods

### 2.1 Fungal strain and culture conditions

Two *H. erinaceus* strains, “Jing3” and “911”, which are distinguished by distinct mycelial colony characteristics, were employed as experimental materials. These two strains were sourced from Huafengyuan Edible Fungus Development Co., Ltd., Anhui Province, China (**Supplementary Table 1**). This study was carried out in the Laboratory of the College of Agriculture, Inner Mongolia Agricultural University, from July to December 2024. Potato Dextrose Agar (PDA) medium, with the composition of 200 g/L potatoes, 20 g/L glucose, 2 g/L KH_2_PO_4_, 2 g/L peptone, 1 g/L MgSO_4_, and 22 g/L agar, was utilized for strain activation and subculture. The prepared PDA medium was autoclaved at 121°C and 0.13 kPa for 20 minutes; under aseptic conditions, the sterilized medium was then poured into 90-mm sterile Petri dishes and allowed to cool for subsequent use. The strains were subcultured onto 90-mm Petri dishes containing Potato Dextrose Agar (PDA) medium and incubated at 25°C. After activation, a 5-mm sterile punch was used to excise a mycelial plug from the edge of the mycelial colony on the solid medium; this plug was then re-inoculated at the center of fresh PDA medium, followed by incubation at 25°C in the dark. Mycelial growth initiated after 3d of incubation, and this time point was designated as the starting point for sampling. Subsequently, mycelium samples were collected at 7d and 14d post-incubation using sterile forceps. These samples were blotted dry on filter paper, immediately snap-frozen in liquid nitrogen, and thereafter stored at -80°C until further use. Each growth stage included three biological replicates.

### 2.2 Measurement methods

Comparative observations and systematic documentation were conducted on the mycelial growth vigor of different strains, including germination time, time to full plate colonization, mycelial growth rate, mycelial density, mycelial color, and colony morphological characteristics.

The mycelial growth rate was determined via the cross method. Specifically, a cross was drawn with the fungal block as the origin, and observations were initiated upon mycelial germination, with the intersection point marked as the growth starting line. Subsequently, perpendicular lines were drawn at the colony margins at 7 and 14d post mycelial growth, and the distance between the two intersection points was measured using a vernier caliper. The average growth rates for the first 7d period and the subsequent 7d period were calculated using the following formula: Average Mycelial Growth Rate (mm/d) = Colony Growth Length (mm)/Culture Days (d).

For each strain, three plates were randomly selected to determine the average biomass yield. Subsequently, the mycelium was scraped off the plates and weighed to quantify the biomass yield (g).

Measurements of protein, soluble sugar, proline contents and cellulase activity were performed with a commercial assay kit (Grace Biotechnology, Suzhou, China) following the manufacturer’s standard protocols. The catalogue numbers: Cat. No. G0417W (protein), Cat. No. G0501W48 (soluble sugar), Cat. No. G0111W48 (proline), and Cat. No. G0709W48 (cellulase).

### 2.3 RNA extraction, transcriptome sequencing, and data analysis

Total RNA was extracted from the mycelia of strains “Jing3” and “911” at different growth stages via the RNA Extraction Kit (RC401-01; Novazenz, Nanjing, China). Three biological replicates were set up for each strain at each growth stage, resulting in a total of 12 samples that were subjected to subsequent transcriptome analysis. RNA quality, concentration, and integrity were evaluated in accordance with the protocol established (Stark et al., 2019). The extracted total RNA was aliquoted and stored at -80°C in an ultra-low temperature freezer for subsequent use.

Upon completion of sample testing, library construction was initiated as follows: (1) mRNA was enriched using Oligo(dT)-conjugated magnetic beads, exploiting the binding between poly(A) tails and Oligo(dT). (2) mRNA was fragmented by adding a specialized buffer to generate segments suitable for cDNA synthesis. (3) Using fragmented mRNA as a template, first- and second-strand cDNA synthesis was performed sequentially. The resultant cDNA was then purified with AMPure XP beads to remove by products. (4) The purified double-stranded cDNA underwent end repair, A-tailing, adapter ligation, and size selection via AMPure XP beads. (5) Finally, PCR amplification enriched the cDNA library for high-throughput sequencing.

Following library construction, the effective library concentration (>2nM) was precisely quantified by Q-PCR to ensure library quality. After quality-assured libraries were obtained, they were pooled according to the target downstream data volume and sequenced on the Illumina platform performed by Shanghai Personal Biotechnology Co., Ltd (Shang hai, China). Following raw read filtering, high-quality clean reads were generated using HISAT2 (v2.2.1) with the latest *H. erinaceus* reference genome (GCA_006506795.2) and default parameters.

Subsequent bioinformatics analysis of the aligned reads proceeded as follows: ANNOVAR was employed for genomic annotation of sequence reads and profiling of read distribution across genomic compartments. Reference-based transcript assembly was performed using StringTie (v1.3.6), and transcript expression levels were quantified via the RSEM 1.2.31 package with FPKM (fragments per kilobase of exon per million mapped fragments) as the expression metric. Principal component analysis (PCA) was employed to evaluate the population distribution of all samples and the stability throughout the analytical process. Meanwhile, Pearson correlation coefficients (PCC) among samples were calculated using the base R cor function and visualized exclusively as heatmaps. Differential expression analysis between comparative groups was conducted using DESeq2, with significant DEGs defined by a *p*-value < 0.05, an absolute fold change ≥ 2, and a false discovery rate (FDR) < 0.05. These samples were divided into four groups (911_7d_VS_911_14d, Jing3_7d_VS_Jing3_14d, 911_7d_VS_Jing3_7d, 911_14d_VS Jing3_14d).

The functional annotation of transcripts was conducted using the phyper package in R software. Two databases were employed for this analysis: the Swiss-Prot database, the Gene Ontology (GO) database, and the Kyoto Encyclopedia of Genes and Genomes (KEGG) database. An E-value threshold of 1×10^^-^5^ was set to filter the annotation results.

### 2.4 Real-time qPCR validation of RNA-Seq data

To validate the differentially expressed loci obtained from RNA-seq data, quantitative real-time PCR (RT-qPCR) was performed. Specifically, 10 DEGs were randomly selected for RT-qPCR analysis to ensure the reliability of the results. The primers for RT-qPCR were designed via Primer Premier 6.0 using the following parameters: melting temperature (Tm) at 58-62°C, PCR product length at 100–300 bp, GC content at 45-55%, and length of primers at 18–25 bp and synthesized by Sangon Biotech Co., Ltd. (Shanghai, China) (**Supplementary Table 2**). The 18S rRNA gene was used as an internal reference gene. The cDNA synthesis was performed with the M-MLV cDNA synthesis kit (Sangon Biotech, Shanghai, China), according to the manufacturer’s instructions. Then, the RT-qPCR reactions were conducted with a Y2xSG Fast qPCR Master Mix (Low Rox) (Sangon Biotech, Shanghai, China) and a QuantStudio5 Real-Time PCR System (ThermoFisher Scientific). Relative gene levels were calculated using the 2^-△△Ct^ method (Trapnell et al., 2013). The verification experiment was performed in three biological replicates, with three technical replicates in each replicate.

### 2.5 Statistical analysis

Statistical analyses were performed using R 4.2.2 software. Data are expressed as mean ± standard deviation (SD). Significant differences between groups were determined by one-way analysis of variance (ANOVA) followed by Tukey’s honestly significant difference (HSD) *post hoc* test, implemented using the Stata (v4.4.2) and agricolae (v1.3-7) packages. Statistically significant differences (*p*<0.05) are indicated by lowercase letters above error bars. The graphical representation were generated with ggplot2 (v3.5.1).

## 3 Results

### 3.1 Mycelial growth characteristics

**Figure 1:**
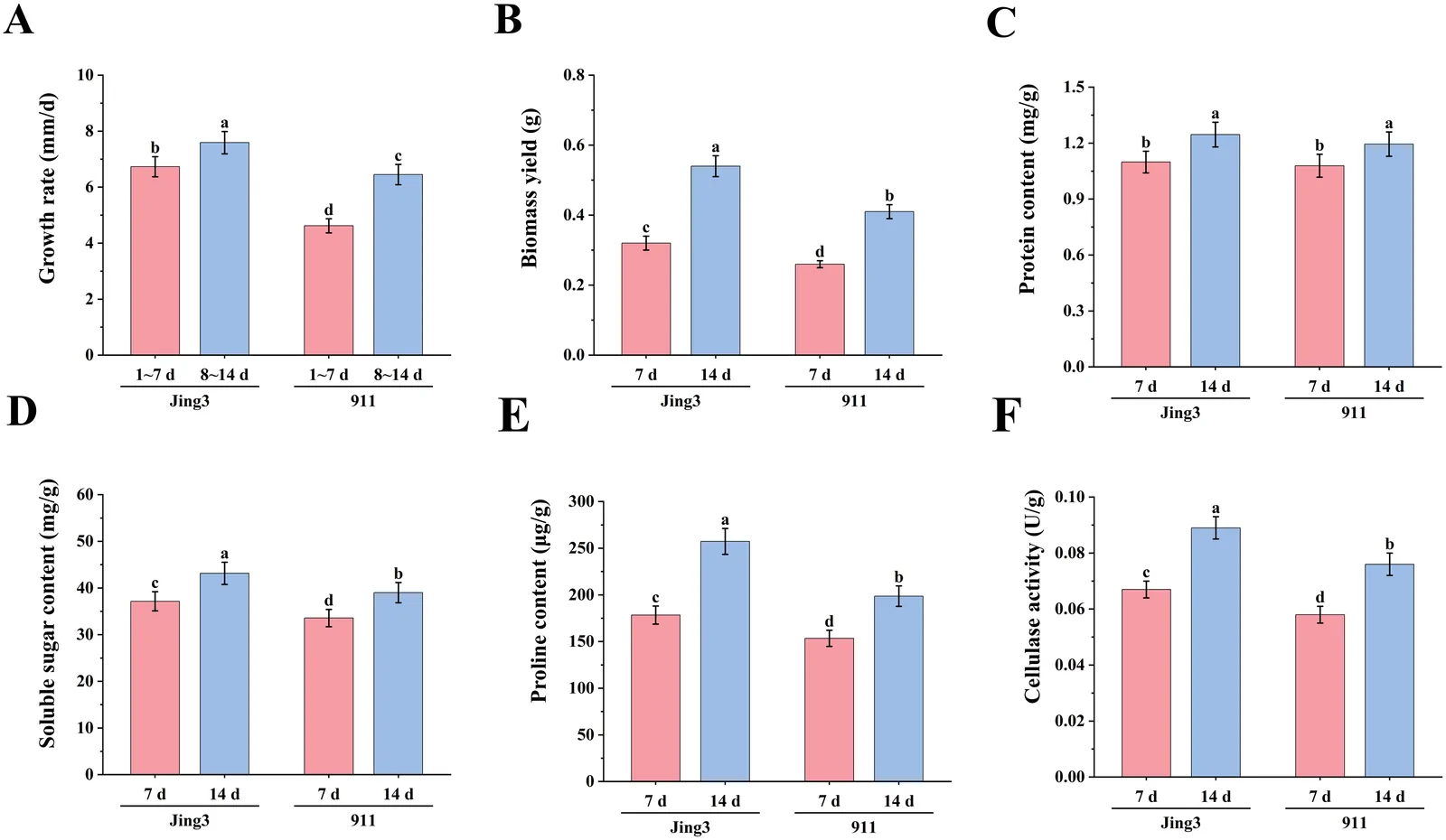
Phenotypic characteristics of different mycelia. **(A)** Growth rate. **(B)** Biomass yield. **(C–E)** Contents of protein, soluble sugar and proline. **(F)** Activities of cellulase.

To elucidate the molecular and physiological mechanisms underlying mycelial growth of *Hericium erinaceus*, the morphological, physiological, and biochemical responses governing mycelial development were systematically assessed in two strains (“Jing3” and “911”) at 7 and 14d (**Figure 1**). Both strains exhibited a consistent germination period of 3d; whereas during subsequent growth stages, the “Jing3” strain displayed enhanced growth vigor and greater mycelial density relative to the “911” strain. Notably, both strains showed significant increases (*p*<0.05) in key growth-related parameters, including mycelial growth rate, biomass accumulation, protein content, soluble sugar content, proline content, and cellulase activity. At both 7 and 14d, the “Jing3” strain significantly outperformed (*p*<0.05) the “911” strain across all assessed parameters. Taken together, these results demonstrate that the “Jing3” strain possesses superior mycelial morphological traits and prominent physiological and biochemical properties compared to the “911” strain. .

### 3.2 Transcriptome analysis

To explore the molecular mechanisms underlying *H. erinaceus* mycelial growth, a comparative analysis was performed on the “Jing3” and “911” strains at two growth stages (7 and 14 days post-mycelial germination). Specifically, a total of 12 libraries were sequenced, with three biological replicates for each stage. All raw RNA-seq datasets have been deposited in the NCBI database under the SRA accession number https://dataview.ncbi.nlm.nih.gov/object/PRJNA1321699?reviewer=qg5rk6d2ubg72agoai1f670ghp. A total of 78.53 Gb of raw reads were generated, and after filtering out low-quality and contaminated reads, 77.09 Gb of clean reads were retained. The Q20, Q30, and GC content of the samples were approximately 98.78%, 96.53%, and 56.87%, respectively (**Supplementary Table 3**).

**Figure 2:**
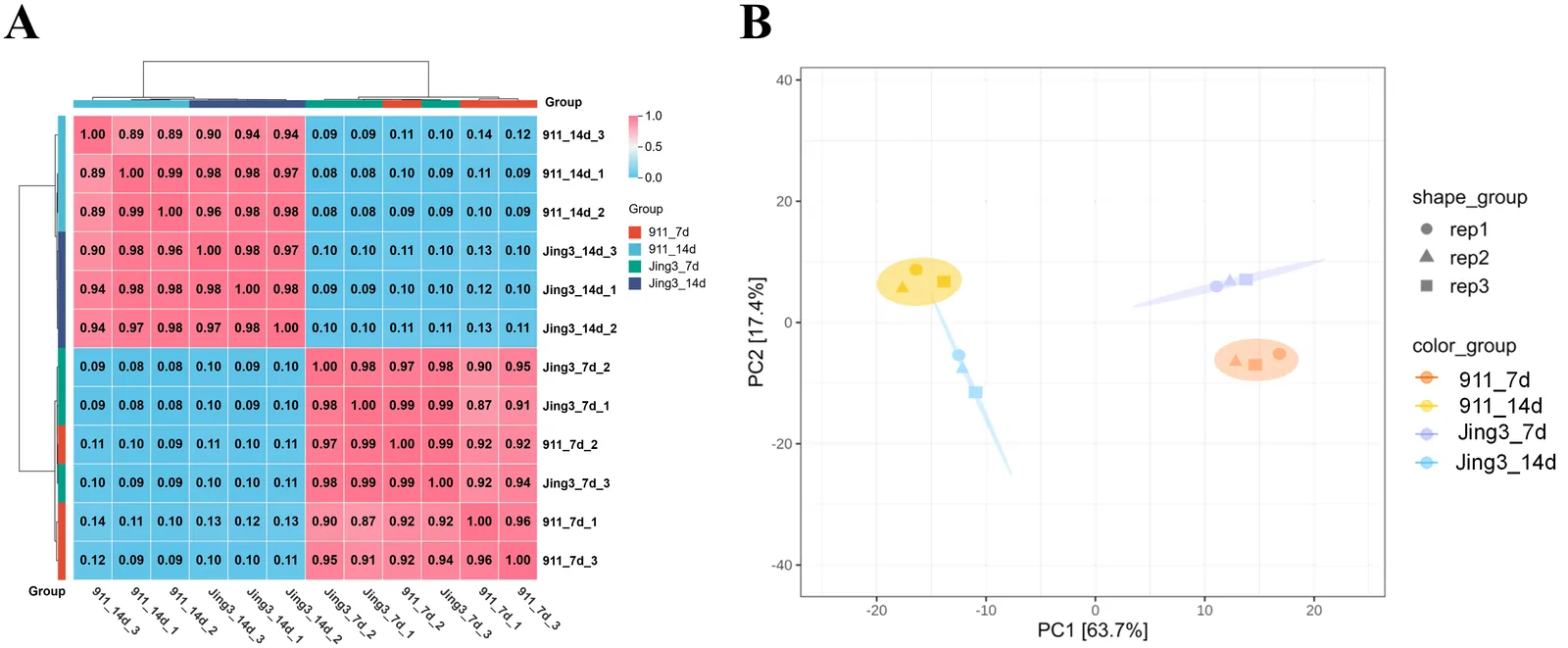
Transcriptome analysis of “911” and “Jing3” mycelia. **(A)** Correlation analysis of 12 samples. **(B)** PCA of 12 samples.

Pearson correlation coefficient analysis of all expressed genes revealed that the correlation coefficients among biological replicates ranged from 0.89 to 1.00, indicating high reproducibility and representativeness of the samples (**Figure 2A**).

Principal component analysis (PCA) was performed on the transcript expression levels of mycelia from the two genotypes (**Figure 2B**). The results showed that samples from the three replicates clustered tightly together, which was consistent with the findings of the correlation analysis. Furthermore, the two genotypes formed distinct clusters at the two growth stages, suggesting significant differences in their regulatory mechanisms underlying growth rate responses.

### 3.3 Differential expression gene analysis

**Figure 3:**
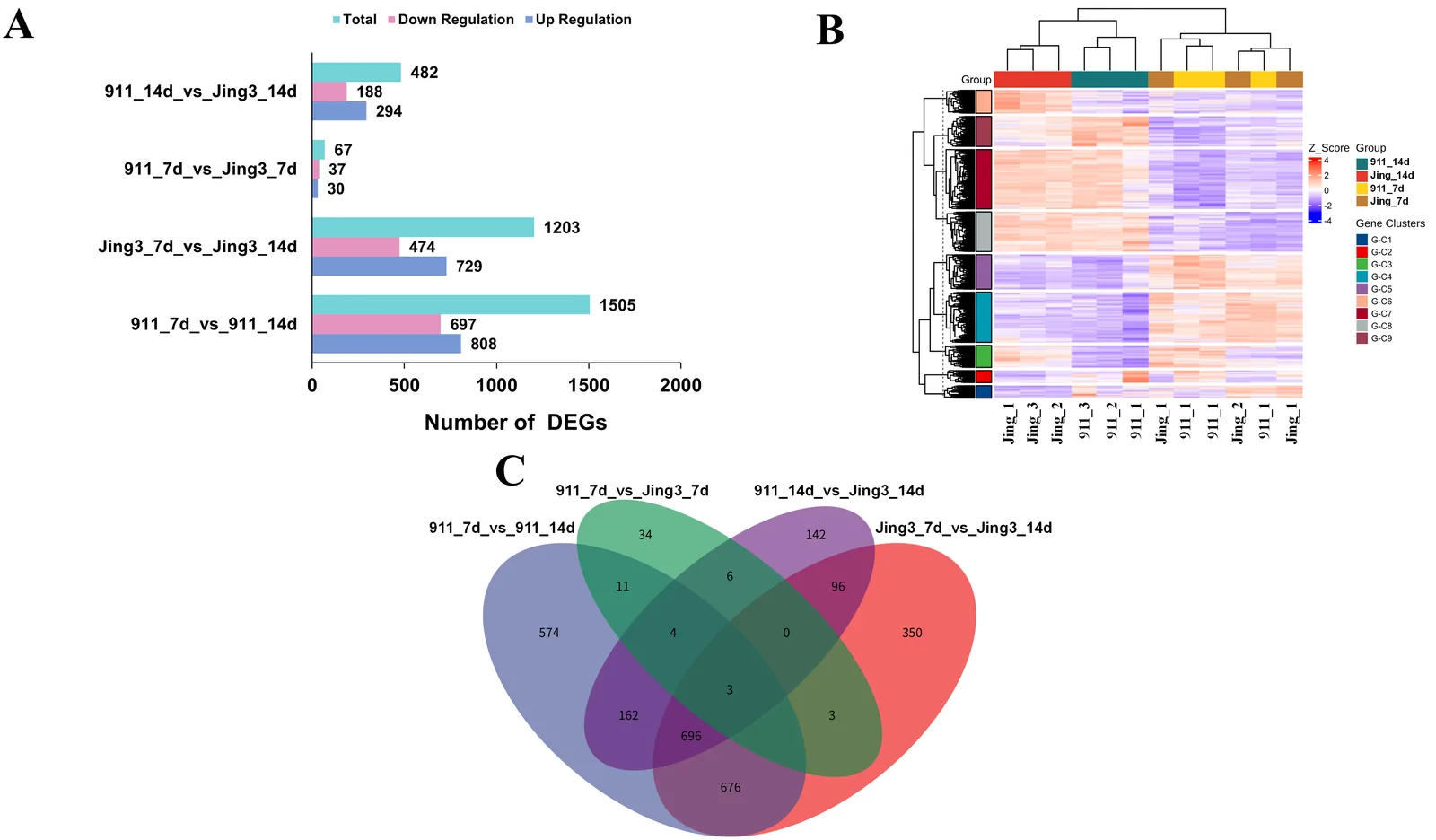
Analysis of differentially expressed genes (DEGs) at different developmental stages. **(A)** The numbers of DEGs in the different comparison groups. **(B)** The heatmap analysis of DEGs in 12 samples. **(C)** Venn’s analysis of the DEGs for different comparison combination in “911” and “Jing3”.

Across the two growth stages, a total of 3,257 differentially expressed genes (DEGs) were collectively identified in the mycelia of the two *H. erinaceus* genotypes (Jing3 and 911) (**Figure 3A**). Specifically, in the 911_7d_VS_911_14d and Jing3_7d_VS_Jing3_14d comparison groups, 1,505 DEGs (808 upregulated and 697 downregulated) and 1,203 DEGs (729 upregulated and 474 downregulated) were identified, respectively. This indicates that the “911” strain exhibits more pronounced transcriptional changes during mycelial growth. Furthermore, in the 911_7d_VS_Jing3_7d and 911_14d_VS_Jing3_14d comparison groups, 67 DEGs (30 upregulated and 37 downregulated) and 482 DEGs (188 upregulated and 294 downregulated) were detected, respectively, suggesting a broader and more complex regulatory response in the late stage of mycelial development. Results from the clustering heatmap indicated that gene expression profiles varied among different mycelial samples across growth phases (**Figure 3B**).

Among all differentially expressed genes (DEGs), 574 were specifically expressed in “911” and 350 in “Jing3”, indicating that the mycelial growth mechanism of “911” is more complex than that of “Jing3” (**Figure 3C**). Additionally, 34 DEGs were uniquely expressed during the 7d phase, while 142 were exclusive to the 14d phase, suggesting that the regulatory mechanisms underlying mycelial growth in the 14d phase are more complex than those in the 7d phase. A total of 676 DEGs were co-expressed in both “911” and “Jing3”, implying significant transcriptional overlap in the genes regulating growth rate between the two genotypes. In contrast, only six DEGs were co-expressed across both phases.

**Figure 4:**
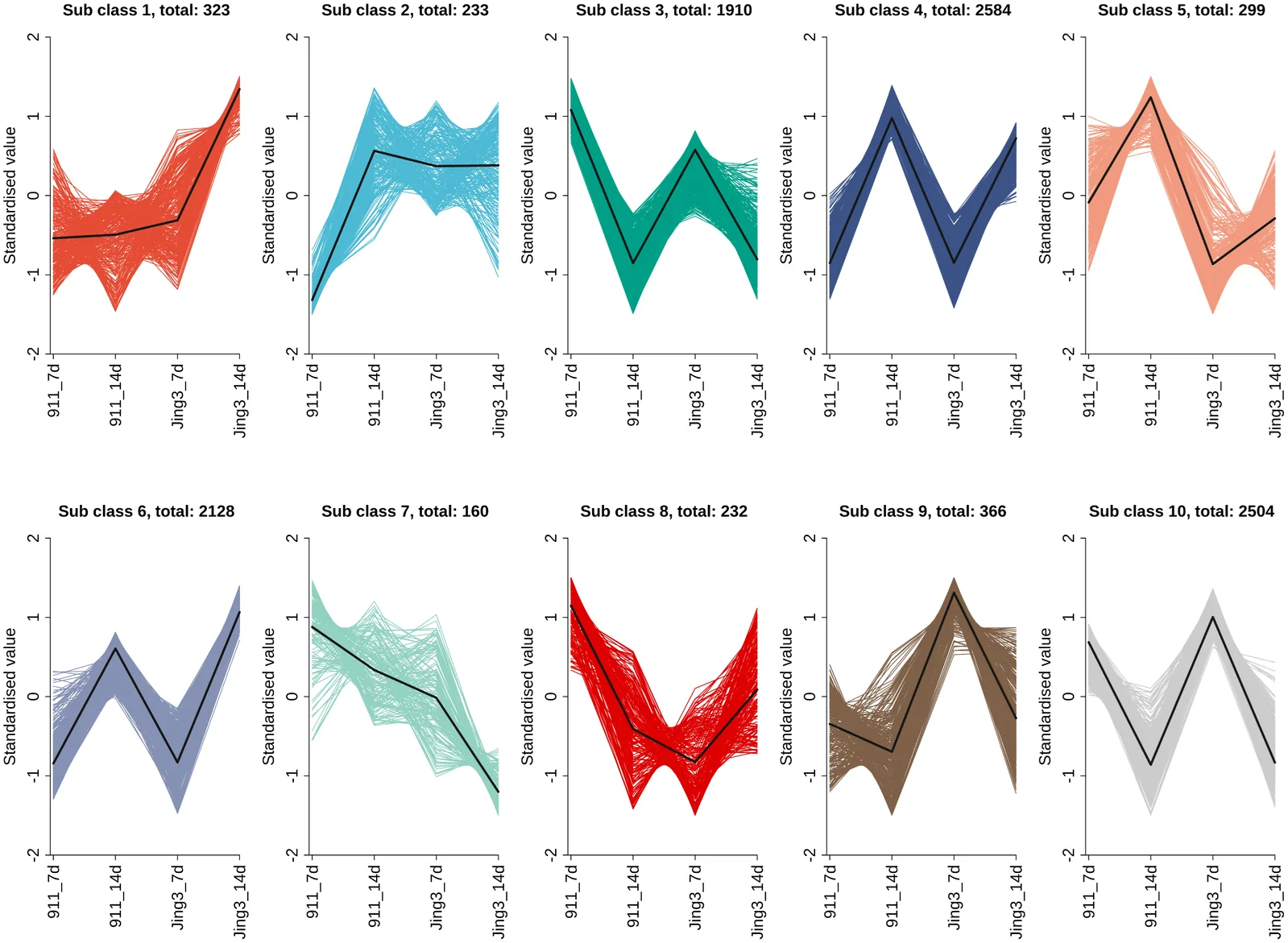
Mfuzz clustering of gene expression at different times.

Mfuzz analysis was used to cluster the expression patterns of differentially expressed genes (DEGs) into 10 clusters. Clusters 1, 4, 5, and 6 exhibited an upward trend in expression levels during mycelial growth in both “911” and “Jing3”, containing 323, 2,584, 299, and 2,128 DEGs, respectively. In contrast, clusters 3, 7, 9, and 10, which comprised 1,910, 160, 366, and 2,504 DEGs, respectively, showed a decreasing trend in expression levels in “911” and “Jing3” as mycelial growth progressed (**Figure 4**).

### 3.4 Functional annotation of DEGs

**Figure 5:**
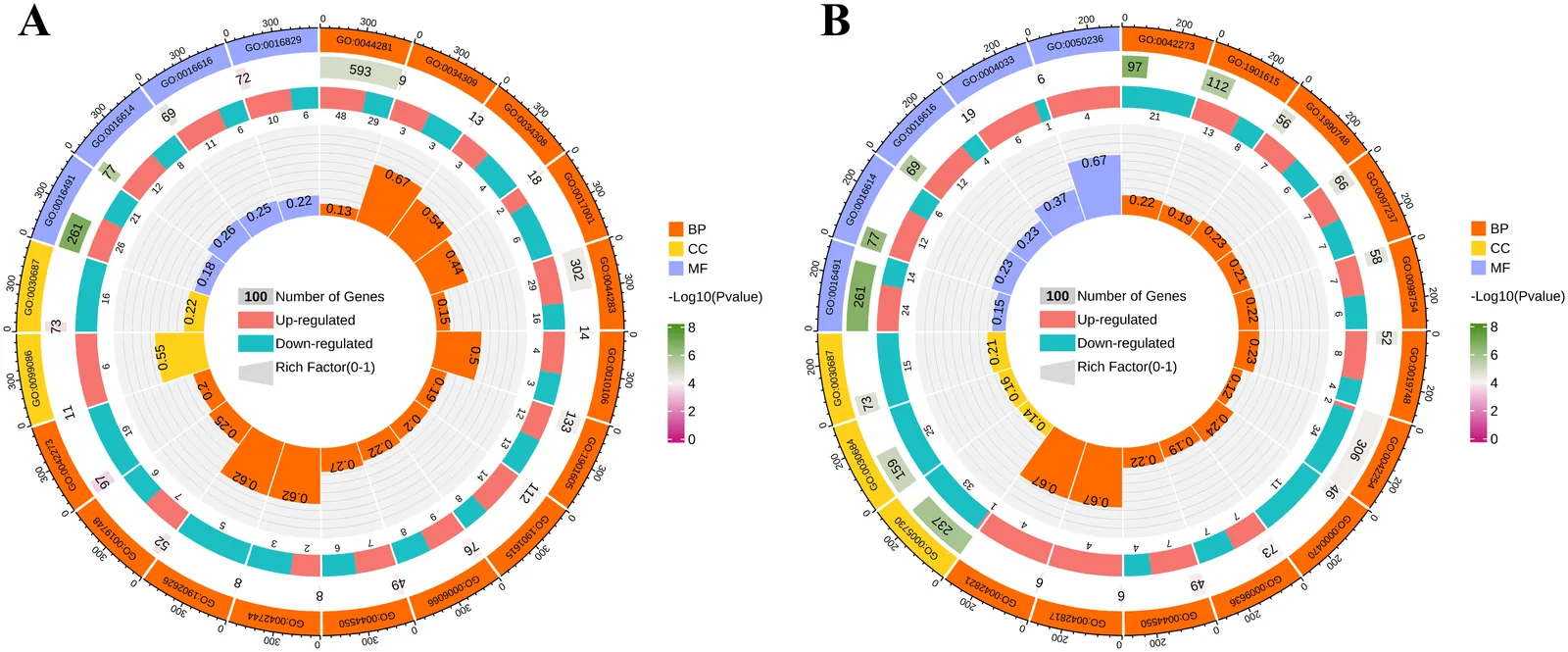
GO enrichment analysis of DEGs. Go enrichment of DEGs of **(A)** “911”, **(B)** “Jing3”.

To clarify functional discrepancies among differentially expressed genes (DEGs), Gene Ontology (GO) enrichment analysis was performed on all DEGs from the two H. erinaceus strains, with a significance cutoff of p<0.05. GO terms were categorized into three major classes: biological process (BP), molecular function (MF), and cellular component (CC). The top 20 most significantly enriched terms are illustrated in **Figure 5**, **Supplementary Tables 4**, **5**, including oxidoreductase activity (acting on the CH-OH group of donors, with NAD or NADP as acceptor), ribosomal large subunit biogenesis, organic hydroxy compound metabolic process, preribosome large subunit precursor, secondary metabolic process, and secondary metabolite biosynthetic process. All these terms are co-enriched in both “911” and “Jing3” strains. In strain “911”, 26.01% of DEGs were enriched in small molecule metabolic process, 15.88% in oxidoreductase activity, and 15.20% in small molecule biosynthetic process. In strain “Jing3”, the top enriched terms were oxidoreductase activity (17.51%), ribosome biogenesis (16.59%), and nucleolus (15.67%).

**Figure 6:**
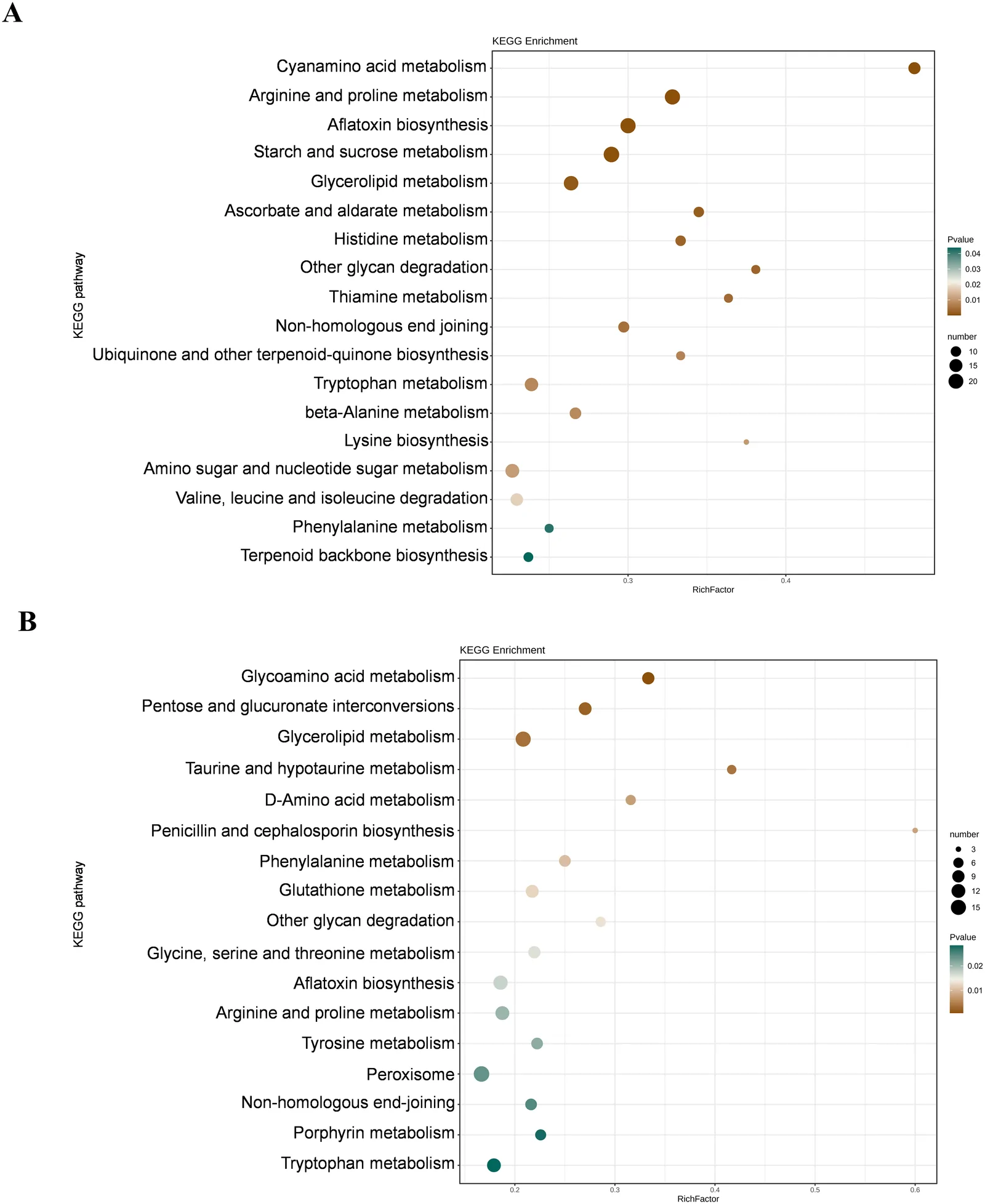
KEGG enrichment analysis of DEGs. KEGG enrichment of DEGs of **(A)** “911”, **(B)** “Jing3”.

KEGG enrichment analysis results (**Figure 6**; **Supplementary Tables 6**, **7**) revealed that differentially expressed genes (DEGs) were significantly upregulated in multiple pathways during mycelial growth of both “911” and “Jing3” strains. These included Cyanoamino acid metabolism, Arginine and proline metabolism, Aflatoxin biosynthesis, Glycerolipid metabolism, Other glycan degradation, Non-homologous end-joining, Tryptophan metabolism, and Phenylalanine metabolism-with the latter commonly enriched in both strains. In strain “911”, 5.45% of DEGs mapped to Arginine and proline metabolism, while 5.20% were enriched in both Aflatoxin biosynthesis and Starch and sucrose metabolism. In strain “Jing3”, the top enriched pathways were Peroxisome (5.10%), Glycerolipid metabolism (4.78%), and Aflatoxin biosynthesis (4.14%).

### 3.5 Analysis of genes related to mycelial growth and amino acid metabolism

**Figure 7:**
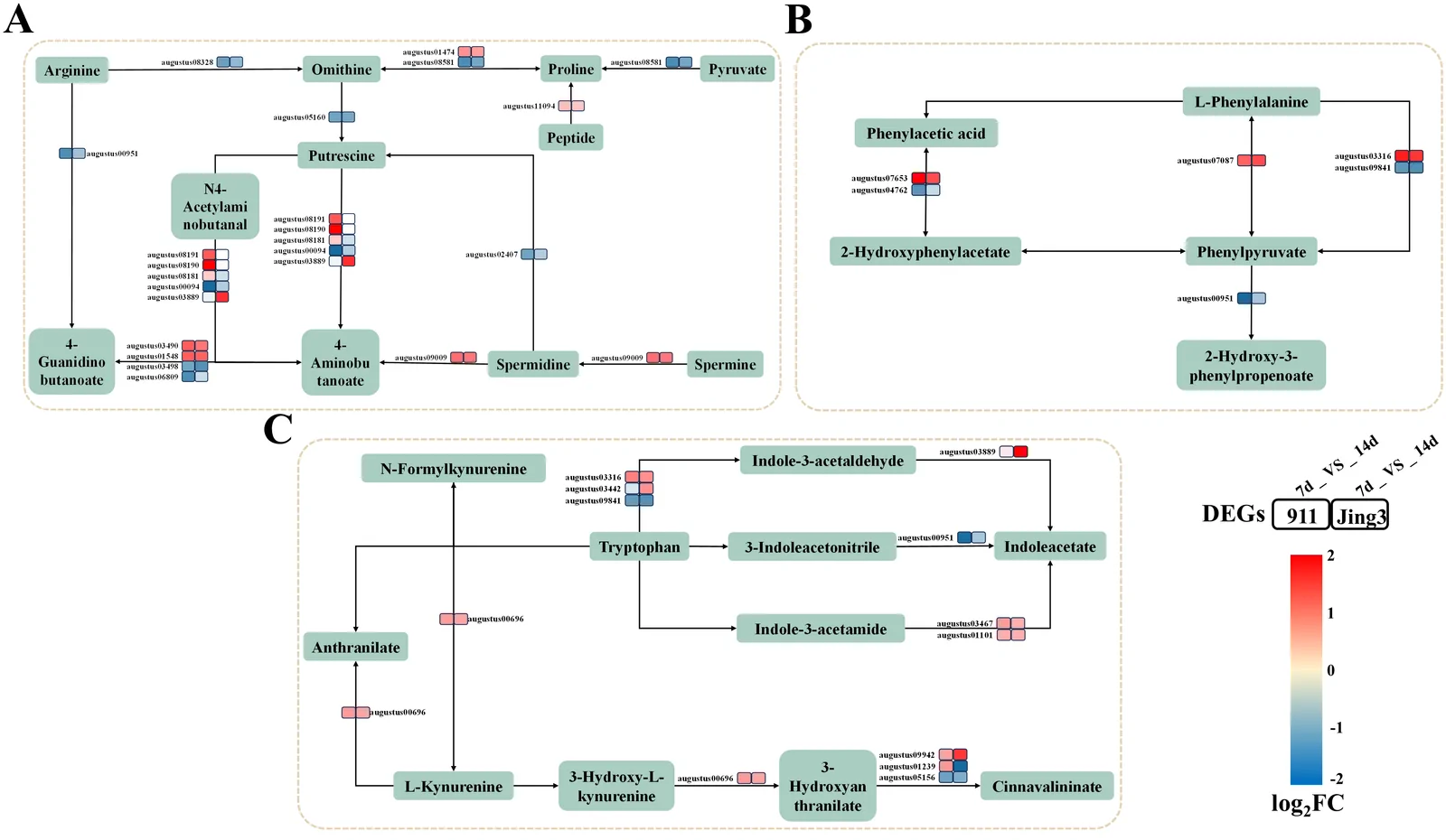
The major genes involved in hyphal growth during amino acid metabolism. **(A)** Arginine and proline metabolism. **(B)** Phenylalanine metabolism. **(C)** Tryptophan metabolism.

KEGG pathway analysis indicated that differentially expressed genes (DEGs) during mycelial growth in “911” and “Jing3” were primarily enriched in amino acid metabolism pathways. To further clarify the mechanisms regulating *H. erinaceus* mycelial growth rate, the expression of genes involved in the three aforementioned pathways was analyzed (**Figure 7**). The most of the 22 DEGs enriched in the “Arginine and proline metabolism” pathway were significantly upregulated in both strains, with a greater number of upregulated genes in “911” compared to “Jing3”. Among the six DEGs enriched in the “Phenylalanine metabolism” pathway, each strain exhibited three upregulated and three downregulated genes. For the 11 DEGs enriched in the “Tryptophan metabolism” pathway, *august03889* and *august09942* were upregulated and displayed high expression levels in “Jing3”. These analyses of amino acid metabolic pathways confirmed that amino acids play a crucial role in mycelial growth.

### 3.6 RNA-seq data validation by RT-qPCR

**Figure 8:**
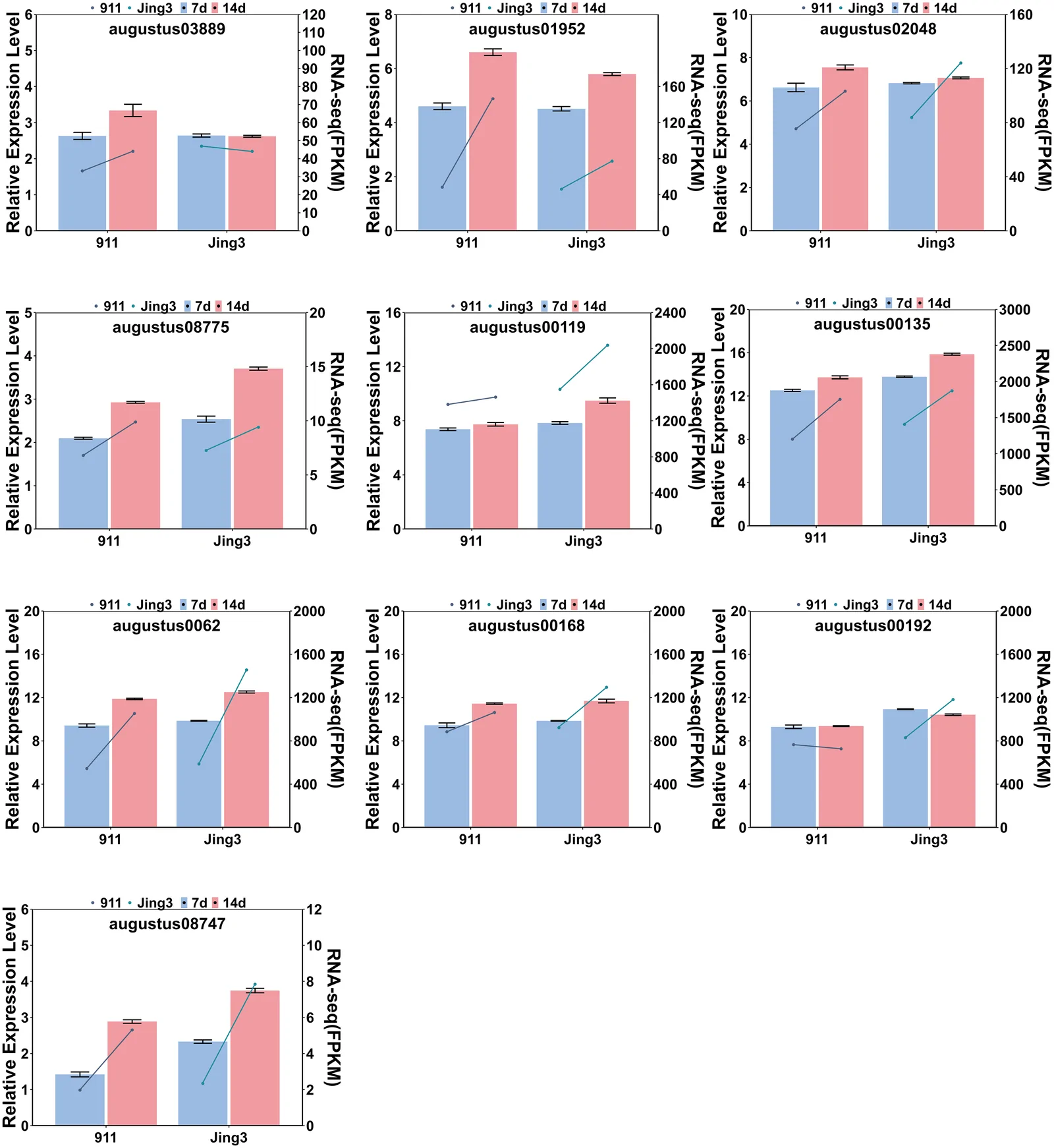
qRT-PCR validation of gene expression levels. Bar graphs (with standard errors) represent the log_2_ fold change of gene expression determined by qRT-PCR (2^-ΔΔCt^ method), and broken lines represent the log_2_ fold change calculated from RNA-seq FPKM values, corresponding to the left and right y-axes, respectively. All assays were performed with three independent biological replicates (n=3). Different lowercase letters above the bars indicate significant differences at *P*<0.05.

To validate the accuracy and reliability of the RNA sequencing (RNA-seq) data, quantitative real-time polymerase chain reaction (RT-qPCR) was performed on ten randomly selected genes (**Figure 8**). The RNA-seq and RT-qPCR datasets exhibited high consistency in the relative expression levels of the selected genes. These results confirm the credibility and accuracy of the RNA-seq data, thereby supporting their suitability for candidate gene screening in subsequent functional studies.

## 4 Discussion

The physiological maturation stage of mycelia in large edible mushrooms is critical for the growth and development of fruiting bodies (Zhang et al., 2023). Only mature mycelia can successfully form primordia and develop into complete fruiting bodies upon stimulation by low temperatures and light (Okuda, 2022). *H. erinaceus* is rich in bioactive substances and exhibits diverse physiological functions. In practical cultivation, *H. erinaceus* requires stringent environmental conditions, making fruiting induction challenging and resulting in relatively low yields. In contrast, *H. erinaceus* mycelia are relatively easy to propagate. Therefore, using *H. erinaceus* mycelia as experimental materials for transcriptomic sequencing and gene regulation studies can enhance experimental controllability and improve the feasibility of subsequent experiments (Huang et al., 2025).

Transcriptomic studies on fungal mycelia have demonstrated that genes related to cell wall and membrane stability, soluble sugars, protein biosynthesis, metabolic pathways, calcium signaling, and mitogen-activated protein kinase (MAPK) pathways play a crucial role in responses to abiotic stress (Fu et al., 2016; Yang et al., 2018). Transcriptomic analyses during fruiting body development have identified differentially expressed genes (DEGs) involved in morphogenesis and primary carbohydrate metabolism, suggesting their potential association with fruiting body formation. However, transcriptome-based investigations into the molecular mechanisms underlying mycelium maturation remain lacking. In the present study, comparative transcriptomics was employed to explore differential gene expression and the molecular mechanisms involved in mycelium maturation (**Supplementary Table 3**). A total of 77.09 Gb of clean reads were generated from comparative transcriptomic analysis of mycelia from two genotypes at different growth stages, with individual samples yielding 5.72-7.42 Gb. These results are consistent with those reported by Zhang et al. in their study on *H. erinaceus* mycelia, confirming the reliability of the transcriptomic data in this study (Zhang et al., 2019).

GO enrichment analysis of differentially expressed genes (DEGs) revealed significant enrichment in terms related to ribosomes and oxidoreductase activity, which aligns with the characteristic rapid growth and nutrient accumulation during mycelium maturation. Ribosomes, ribonucleoprotein complexes present in all living cells, function as the molecular machinery for protein synthesis in organisms (Meng et al., 2021). In the present study, GO enrichment analysis identified DEGs enrichment in ribosome-related categories (**Figure 5**), a pattern consistent with the expression profiles observed in *Flammulina velutipes* and *Auricularia* (Nie et al., 2025; Yang et al., 2018). This suggests an increased demand for proteins during hyphal growth and development. Additionally, ribosomes are involved in processes such as DNA repair, organismal development, and cell division (Liu et al., 2022). Reactive oxygen species (ROS), a class of multifunctional key factors, participate in various physiological processes including hyphal growth and cell cycle progression (Fu et al., 2017; Yang et al., 2021). In *Podospora anserina*, ROS accumulation triggers mitochondrial-dependent programmed cell death (PCD), which is recognized as the core mechanism underlying biological aging in this fungus (Osiewacz and Schürmanns, 2021). This further explains the induction and activation of certain aging-related genes during the growth and development of *H. erinaceus* mycelia.

KEGG enrichment analysis revealed that the differentially expressed genes were primarily associated with pathways involved in nucleotide synthesis and amino acid metabolism (**Figure 6**). Similar metabolic patterns have been reported in studies on *Phellinus linteus* mycelium (Chen et al., 2019). In mature *Hypsizygus marmoreus* mycelium, metabolites related to pyrimidine synthesis, such as cytidine monophosphate (CMP) and cytidine, were significantly enriched, indicating a high demand for rapid nucleotide synthesis during active growth (Xiang et al., 2023). As essential nutrients, amino acids serve as both organic nitrogen sources that promote fungal growth and as growth factors regulating metabolic activity (McCarthy and Walsh, 2018). Investigations into amino acid metabolism across different developmental stages of *H. marmoreus* demonstrated a close correlation between gene expression profiles and amino acid metabolic processes, implying that amino acids may play a role in the physiological maturation of the mycelium (Yang et al., 2021). Likewise, significant variations in amino acids and their derivatives have been observed throughout various nutritive growth phases in *H. erinaceus* mycelium. Collectively, these findings suggest that amino acid biosynthesis and metabolism are critically involved in the physiological maturation of most mushroom mycelia.

Proline reduces reactive oxygen species (ROS) levels in edible fungi and inhibits programmed cell death (Xu et al., 2022). Arginine regulates organismal growth and development via its metabolites and associated enzymes (Yang et al., 2021; Osiewacz and Schürmanns, 2021). Phenolic compounds derived from phenylalanine metabolism, such as gallic acid, possess antioxidant properties and can scavenge excess intracellular ROS (Liu et al., 2019). Tryptophan exhibits antioxidant activity due to its unique -OH and -NH functional groups (Gong et al., 2022). In the present study, analysis of the expression levels of genes involved in phenylalanine, tryptophan, arginine, and proline biosynthesis revealed distinct expression trends (**Figure 7**). These findings indicate that the mycelium of *H. erinaceus* may actively respond to reactive oxygen species (ROS) generated during growth, thereby promoting mycelial development.

Several limitations of this study should be noted. This work only involved two *H. erinaceus* genotypes and two growth stages, which is insufficient to fully resolve the complete transcriptional dynamics of mycelial physiological maturation, and the conclusions need to be further verified in more strains. In addition, functional inferences in this study are mainly based on transcriptomic data and homologous annotation, lacking direct experimental evidence. In follow-up studies, we will expand the number of strains and developmental stages, carry out systematic co-expression network analysis, and combine multi-omics and molecular functional verification to deeply explore the regulatory mechanism of *H. erinaceus* mycelial maturation.

## 5 Conclusions

This study elucidated the molecular mechanisms underlying mycelial growth in two H. erinaceus strains (Jing3 and 911) through RNA sequencing analysis at 7 and 14 days post-mycelial germination. Global transcriptomic profiling identified 3,257 differentially expressed genes (DEGs), revealing that the 911 strain exhibited more pronounced transcriptional dynamics during growth and a more complex regulatory mechanism compared with Jing3, as indicated by the greater number of strain-specific DEGs. Further interstage comparisons confirmed that late-phase mycelial development involves a more intricate regulatory network. GO and KEGG enrichment analyses highlighted the conserved roles of oxidoreductase activity, ribosomal biogenesis, and secondary metabolism in both strains, while pinpointing amino acid metabolism pathways as core regulatory factors governing mycelial growth. The genotype-specific expression patterns of amino acid metabolic pathways further accounted for the divergent growth characteristics between the two strains. Collectively, these results provide valuable molecular insights into the genetic improvement and cultivation optimization of *H. erinaceus.*

## Data Availability

The original contributions presented in the study are publicly available. This data can be found here: NCBI Sequence Read Archive, accession PRJNA1321699.
